# The Role of Neurosurgical Techniques in Management of Acute and Chronic Stroke: A Comprehensive Literature Review

**DOI:** 10.7759/cureus.65671

**Published:** 2024-07-29

**Authors:** Yiorgos Antoniadis, Sana A Khan, Sandhya Nallamotu, Akash Ranganatha, Jessamine Edith S Ferrer, Gargi Gautam, Jade Todras, Renée Campbell, Suresh Chelluri, Naga M Parvathaneni

**Affiliations:** 1 Department of Medicine, St. George's University School of Medicine, Saint George, GRD; 2 Department of Surgery, Liaquat College of Medicine and Dentistry, Karachi, PAK; 3 Department of Surgery, Kasturba Medical College of Manipal, Manipal, IND; 4 Department of General Surgery, Murrieta Valley Surgery Associates, Wildomar, USA; 5 Department of Surgery, Jagadguru Jayadeva Murugarajendra Medical College, Davangere, IND; 6 Faculty of Medicine and Surgery, University of Santo Tomas, Manila, PHL; 7 Department of Internal Medicine, Georgian National University SEU, Tbilisi, GEO; 8 Department of Biology, Suffolk County Community College, New York, USA; 9 Department of Internal Medicine, St. George’s University, Saint George, GRD; 10 Department of Surgery, Rajiv Gandhi Institute of Medical Sciences, Telangana, IND; 11 Department of Surgery, International Higher School of Medicine, Bishkek, KGZ

**Keywords:** clot retrieval, decompressive, endovascular, hemicraniectomy, neurosurgical interventions, revascularization surgery, stereotactic surgery, stroke treatment

## Abstract

Stroke is a medical condition that results from a decreased or completely diminished supply of blood to the brain, and it is considered one of the major causes of morbidity and mortality globally. Stroke is categorized as ischemic and hemorrhagic stroke, both of which demand prompt and particular timely intervention. This extensive review is done to investigate the precise management of acute and chronic manifestations of stroke in relation to neurosurgical interventions, ultimately providing a thorough analysis regarding indications, procedures, outcomes, and complications that are associated with it. In this regard, a pervasive review of literature was carried out, which was primarily sourced from literature databases such as PubMed. This paper particularly outlines a sound relative analysis of anticipating the competence of each neurosurgical technique in use. Endovascular clot retrieval (ECR) has been particularly highlighted, as its effectiveness has been profoundly observed when selected as a treatment option within a time period of 6-24 hours following an ischemic stroke. In less than a time frame of 48 hours, decompressive hemicraniectomy (DH) is usually considered the most suitable treatment for cases of intracranial hypertension resulting from middle cerebral artery (MCA) infarction. Hemorrhages that occur due to ruptured aneurysms are most commonly dealt with clipping and neuroendovascular techniques. Additionally, considering that revascularization surgery is time-sensitive, the results can ultimately vary. Competent results have been linked with stereotactic surgery, which includes deep brain stimulation (DBS) and focused ultrasound ablation (FUSA), which are also famous for being minimally invasive in nature. However, the broader application of these techniques is hindered by the absence of established protocols. This review highlights the importance of timely interventions, advanced equipment, and precise medical protocols to optimize treatment outcomes.

## Introduction and background

Worldwide, cerebrovascular accidents and stroke are the second leading cause of death and the third leading cause of disability [1]. Stroke, the sudden death of some brain cells due to lack of oxygen when the blood flow to the brain is lost by blockage or rupture of an artery to the brain, is also a leading cause of dementia and depression [2]. The first recorded use of “stroke” as a lay term was in 1599, attributing the sudden onset of symptoms to a “stroke of God’s handle” [3]. The word “stroke” is related to the Greek word “apoplexia,” which implies being struck with a deadly blow [4]. Stroke remains one of the most devastating of all neurological conditions [5]. Worldwide, it accounts for approximately 5.5 million deaths annually, with 44 million disability-adjusted life-years lost [5]. As a disease of aging, the prevalence of stroke is expected to increase significantly around the world in the years ahead as the global population older than 65 years of age continues to increase by approximately 9 million people per year [6].

Stroke, a serious medical condition that disrupts brain blood flow, has several causes and effects. A blood clot stops a brain artery, causing ischemic stroke, the most common type. About 87% of strokes are this type [7]. Thrombotic stroke occurs when a clot forms in brain arteries, while embolic stroke occurs when a clot travels to the brain from another body region. When a blood artery ruptures, brain bleeding causes hemorrhagic strokes, which are rare but deadly. These include intracerebral and subarachnoid hemorrhages. Transient ischemic attack (TIA), also known as a mini-stroke, adds to the list. Future strokes are potentially indicated by a temporary blockage of blood supply to the brain. Being aware of the categories mentioned above will serve as a guiding light to recognize the symptoms in a timely manner and bring ease to seeking proper medical help, which will ultimately result in improving and saving more lives.

To reduce the mortality rates, amplification of the functional results, and enhancement of the overall quality of living for those who have survived strokes, systematic management is therefore required for both acute and chronic stroke. Precise therapies, including intravenous thrombolysis and several individual neurosurgical interventions, have a considerable effect on the outcome of patients during the acute phase as they play an important role in re-establishing the blood flow to the brain and minimizing brain tissue damage.

Developing on these substantial research findings, the ultimate goal of this present review paper is to explore the implications of various neurosurgical techniques that are being employed in the management of both acute and chronic stroke.

## Review

Management of acute stroke

Table 1 illustrates the various neurosurgical interventions used in the immediate response to acute stroke, emphasizing techniques like endovascular clot retrieval (ECR) and decompressive hemicraniectomy (DH).

**Table 1:** Acute stroke management ECR, endovascular clot retrieval; LVO, large vessel occlusion; MISTIE, minimally invasive surgery plus alteplase

Management type	Indications and techniques	Complications
ECR	Indicated within 6-24 hours for ischemic stroke with LVO	Hemorrhage, reocclusion, and access site issues
	Techniques include stent retrievers and aspiration devices	
Neurosurgical clipping	For aneurysmal subarachnoid hemorrhage	Rebleeding, brain edema, and infarction
	Techniques: open craniotomy, placement of clips on aneurysm neck	
Mechanical thrombectomy	Techniques: coiling, clot aspiration, and MISTIE	Hemorrhagic issues and infections
	Aim: reduce hemorrhage volume and enhance functional recovery	
Decompressive hemicraniectomy	Indicated for severe ischemic strokes with brain swelling	CSF disturbances, seizures, and syndrome of the trephined
	Procedure: removal of part of the skull to relieve pressure	

Endovascular clot retrieval 

The preferred treatment in case of acute ischemic stroke (AIS), which happens as a result of arterial large vessel occlusion (LVO), is ECR, which is also known as mechanical thrombectomy (MT). ECR has been significantly associated with decreased rates of morbidity and mortality, but the downside of this is a notably costly treatment option compared to the rest of the conventional treatment methods, as the major cost is shared by the required equipment and staff [8,9]. Promising results of the treatment with ECR have been seen if the procedure is performed within the time frame of 6-24 hours in total, starting from the onset of the particular symptoms in patients with substantial core values for ischemia [10,11].

Major arterial blockage, along with the assessment of infarct core and penumbra size, has been primarily identified by using CT angiography and non-contrast enhanced CT scans. For the same purpose, MRI and DSA can also be opted as diagnostic tools, but accessibility problems make MRI a less commonly used parameter [12]. Instruments like stent retrievers, aspiration devices, and balloon-guiding catheters are majorly utilized in ECR. The thrombus is reached through femoral artery puncture by inserting a balloon-guided catheter. A microcatheter is then inserted over the thrombus when it is penetrated with a guidewire, followed by the opening of the stent, which grasps the thrombus, and finally, it is withdrawn back in addition to the microcatheter [13,14].

An alternative method that is used to remove the thrombus is performed with the help of a large-bore catheter, and it results in direct aspiration of the thrombus, known as the direct aspiration first pass technique (ADAPT) [15]. This method demonstrates the evolving nature of MT.

Regulating blood pressure is crucial to prevent subsequent bleeding, with a target of less than 185/110 mmHg initially post-ECR. Anticoagulation may be necessary immediately after clot extraction, depending on individual patient circumstances [16,17].

Potential complications include symptomatic intracerebral hemorrhage (ICH), stenosis at the thrombectomy site, vessel perforation and dissection, hematomas, and reocclusion [18,19]. Despite these risks, ECR is highly effective, particularly for anterior circulation strokes, where 40% of patients achieve functional independence at three months compared to 25% with optimal medical care [20]. For posterior circulation strokes, outcomes are more variable [21]. Prompt and safe application of MT, along with interprofessional communication and efficient workflows, are essential for patient success [22,23].

MT effectively removes large clots, minimizing long-term disability in AIS patients. However, it is associated with risks such as access site complications, device-related issues, and other MT-related complications [24]. Preventive measures and early detection are vital for minimizing these risks. Since 2015, MT has been the standard treatment for patients with AIS meeting specific criteria [25]. Studies have shown favorable outcomes for patients with low Alberta Stroke Program Early Computed Tomography Scores (ASPECTS) undergoing MT, highlighting the importance of successful recanalization [26].

Neurosurgical clipping for hemorrhagic stroke

Subarachnoid hemorrhage is a subgroup of hemorrhagic stroke that occurs approximately in nine out of 100,000 people per year. Among the incidence of subarachnoid hemorrhage, 85% is due to a ruptured intracranial aneurysm. Aneurysmal subarachnoid hemorrhage survivors are vulnerable to rebleeding until the occlusion of aneurysm is achieved. In hemorrhagic stroke, particularly in aneurysmal subarachnoid hemorrhage, widely accepted interventions to obliterate the blood flow in the aneurysm include surgical clipping and endovascular coiling [27].

According to a study done by Sharma et al., the indications that are favorable to microsurgical clipping include the young age group of <40 years old or <70 years old; the presence of intracranial hematoma; wide neck aneurysm; aneurysms located in the middle cerebral artery (MCA) and pericallosal artery; large aneurysm of size >4 mm or >10 mm; and in the setting of insufficient endovascular access, intra-aneursymal thrombus, arterial branch occlusion, atheromatous, and fibromuscular dysplasia, when complete occlusion is unlikely and during implantation of stent and balloon remodeling [28].

Neurosurgical clipping is done under general anesthesia. Open surgery, particularly craniotomy, is a prerequisite before placing the clips. Once the skull is open, gentle retraction of the brain parenchyma is performed to expose the aneurysm. A small titanium clip is then permanently placed across the neck of the aneurysm to prevent blood circulation into it [29].

Neurosurgical clips are more durable and decrease the retreatment rate of patients. In the meta-analysis study of Zhu et al., the benefits associated with surgical clipping are total occlusion of aneurysms and low risk of rebleeding [30]. Concerning postoperative rebleeding up to one year, the relative risk of rebleeding was lower for the neurosurgical clipping treatment group at 1.4% (17/1137) versus the endovascular coiling group at 2.6% (32/1229) [27].

Significant changes in cerebral circulation and poorer prognosis were observed in neurosurgical clipping, with higher rates of delayed cerebral ischemia, poorer functional outcomes, and increased mortality compared to endovascular coiling over one, five, and 10-year follow-ups [27].

The complications of neurosurgical clipping may include a hemorrhagic event, brain swelling edema, infarction, hypotension, and cardiac arrhythmias [29]. In a study by Lee et al., complications sustained from the clipping intervention in 50 patients include aneurysm rebleed (6%), delayed hydrocephalus needing ventriculoperitoneal shunt (40%), delayed ischemic neurologic deficit (8%), large infarction requiring DH (22%), venous thromboembolism (2%), and in-hospital mortality (20%) [31].

Another study done by Chen et al. revealed that several complications were seen in 172 patients allocated to the neurosurgical clipping modality [32]. These include in-hospital mortality (0.6%), cerebral infarction (7%), hydrocephalus (9.3%), subdural hematoma (1.2%), oculomotor nerve palsy (3.5%), intraoperative rupture (3.5%), decreased sight (0.6%), and rebleeding (1.2%) [32].

Minimally invasive endovascular procedures

Minimally invasive endovascular procedures, such as neuroendovascular coiling, clot aspiration, and minimally invasive surgery plus alteplase (MISTIE), offer promising treatment options for ICH and other cerebrovascular conditions. Neuroendovascular coiling involves the insertion of coils to occlude aneurysms, preventing rebleeding and promoting healing [33]. Clot aspiration techniques, like those utilized in the MISTIE procedure, involve the removal of clots through small cranial openings, minimizing tissue disruption and potentially improving functional outcomes [34].

The MISTIE III trial revealed that if the least invasive surgery is used in combination with thrombolysis, this approach can remarkably decrease the volume of clot and perihematomal edema, which will undoubtedly result in the improvement of long-term functional consequences in patients with ICH [35]. Decreased mortality, along with enhanced clinical outcomes in patients, has been linked to decreased perihematomal edema and profound clot resolution by these procedures [36]. Recent studies have also highlighted that the favorable outcome of the minimally invasive treatment options is largely dependent upon surgical performance along with accurate catheter placement [36].

Decompressive hemicraniectomy 

DH is opted as a treatment option when complications such as brain swelling and increased intracranial pressure emerge as a result of cerebral infarctions [37]. It falls under the category of life-saving therapy for malignant MCA infarctions, which is generally associated with a higher risk for mortality, being 80% [38]. The specific procedure targets the reduction of intracranial pressure, alleviates brain compression, and promotes brain tissue perfusion [39]. The American Heart Association/American Stroke Association guidelines recommend decompressive craniectomy within 48 hours from cerebral edema regardless of medical intervention for patients specifically ≤60 years old along with neurological decline [40].

DH involves removing a segment of the skull to allow the brain to expand outward, reducing intracranial pressure [39]. The common technique is the frontal-temporal-parietal DH. The patient is positioned supine with the head turned away from the affected side. A wide curvilinear incision is made, and the scalp and temporalis muscle are retracted. A bone flap is removed, and a dural opening is achieved by durotomy. An expansive duraplasty is performed using autologous grafts, and the wound is closed with sutures [41,42].

Randomized controlled trials have shown that DH significantly reduces mortality in patients <60 years old with malignant MCA infarctions. For instance, the DECIMAL trial demonstrated a reduction in six-month mortality from 78% to 25% with surgery [41]. The DESTINY trial showed a 30-day mortality reduction from 57% to 12% with surgery [37]. However, patients >60 years old have poorer functional outcomes, with moderate disability mRS scores seen in only 6% of surgical patients [40].

Complications of DH include hemorrhages, infections, cerebrospinal fluid disturbances, seizures, and syndrome of the trephined. Inadequate craniectomy size can lead to external herniation and brain bleeding. Surgical site infections and disorders of cerebrospinal fluid dynamics, such as hygroma and hydrocephalus, are common. Seizure prevalence is higher in patients undergoing this procedure [41,42]. Syndrome of the trephined can occur, causing new neurologic deficits due to sinking scalp pressure on the brain [43]. Functional outcomes are generally poorer in older patients >60 years old, and early or delayed surgery can impact prognosis negatively [37,40].

Neurosurgical techniques for chronic stroke

Table 2 depicts strategies for managing the long-term effects of stroke, focusing on revascularization methods such as carotid endarterectomy (CEA) and extracranial-intracranial (EC-IC) bypass and neuromodulation techniques like deep brain stimulation (DBS) and focused ultrasound ablation (FUSA).

**Table 2:** Chronic stroke management EC-IC, extracranial-intracranial; DBS, deep brain stimulation; FUSA, focused ultrasound ablation

Management type	Techniques and indications	Complications
Revascularization surgery	Techniques: direct (e.g., EC-IC bypass) and indirect (e.g., carotid endarterectomy)	Stroke during surgery and myocardial infarction
	Indications: chronic ischemic strokes with significant artery stenosis	
Stereotactic surgery	Techniques: DBS and FUSA	Hemorrhage, infection, and targeted area damage
	Indications: motor deficits, pain syndromes, and epilepsy from stroke	

Revascularization surgery

Neurosurgical revascularization effectively treats cerebrovascular diseases, including ischemic strokes, hemorrhagic strokes, and intracranial aneurysms. This technique is classified into direct cerebral revascularization, like EC-IC bypass, and indirect methods, such as CEA [44].

Direct cerebral revascularization, initiated by Yasargil in 1967, primarily uses the superficial temporal artery to MCA (STA-MCA) anastomosis [45]. This approach is especially beneficial for severe ischemic strokes in the MCA territory, improving neurological outcomes when performed early, particularly in patients under 60 years old [45-47].

Indirect revascularization through CEA involves removing atherosclerotic plaque from the carotid artery to enhance cerebral blood flow. This is recommended for patients with symptomatic carotid stenosis who have experienced related symptoms within the last six months and have 70-99% stenosis. This procedure is associated with a reduced perioperative risk of stroke and death if performed under suitable conditions [48,49].

Both techniques aim to restore cerebral circulation and prevent further neurological deterioration. Direct revascularization creates a new pathway for blood flow in affected areas [44]. Cautious exposure of the arteries, an accurate anastomosis, and efforts to avoid complications, like cerebrospinal fluid leakage, are all included in the procedure of STA-MCA bypass [50].

The carotid artery is normally exposed with the help of CEA, which typically positions the patient in a supine position with their head rotated. The surgical procedure normally comprises meticulous dissection, plaque removal, and frequently accomplished closure of the artery to prevent narrowing with a patch [51,52].

Profound risks are generally associated with these surgeries, like perioperative stroke and myocardial infarction, regardless of the benefits. The North American Symptomatic Carotid Endarterectomy Trial (NASCET) authenticates that CEA remarkably decreases the risk of strokes in the future in patients with significant carotid stenosis [53]. Nonetheless, the efficacy of EC-IC bypass to prevent stroke recurrence is inconsistent, and this is the reason that the procedure has now become an infrequent practice in acute settings [54-56].

Altogether, where the net results of revascularization surgeries show improvements in certain patient populations, precise execution is required, and some factors such as the age of the patient, the extent of artery occlusion, and the timing of the intervention are among the list of limiting factors [53,56-59].

Stereotactic surgery 

Advanced imaging techniques such as MRI or CT scans are used as stereotactic surgeries with the least invasive techniques to specifically target particular brain areas. Patients who have had a stroke and have not responded sufficiently to standard medical therapy and rehabilitation may find this surgical procedure beneficial in particular. The procedure guarantees excellent particularity with a minimal number of incisions and centralization of the treatment of stroke-related lesions of the brain by the utilization of complex and advanced virtual planning and imaging technologies [60].

Critical brain areas are precisely mapped with the help of templates and navigation systems in order to enhance perfection and minimize complications, and this technique involves both protocols: static and dynamic guided [60]. The precision and accuracy of these computer-assisted treatment procedures are additionally enhanced by the incorporation of state-of-the-art technologies, such as optoelectronic motion capture systems, minimizing possible errors and ameliorating surgical outcomes [61].

Stereotactic surgeries are indicated for patients with chronic stroke who experience persistent motor deficits, pain syndromes, or intractable epilepsy resulting from stroke-induced brain damage. Fundamental to the present approach are techniques like FUSA and DBS, which target the affected networks of the brain and result in supplementing the potential for motor recovery and symptom management [62].

Deep brain stimulation

DBS has substantially evolved from a treatment option for movement disorders to a possible therapeutic plan for numerous neurological and psychiatric conditions such as stroke, enhancing neuroplasticity and motor recovery [63]. Specific brain areas are stimulated by using implanted electrodes of DBS, targeting functional restoration and pain alleviation in patients with chronic stroke along with severe motor impairments [64].

Patient selection for DBS is quite a strict and thorough process, as it is only opted once all other treatment modalities have been unsuccessful. It involves an in-depth analysis of the symptoms of the patient, all the prior treatments the patient has gone through, and the overall quality of life [63]. Regardless of the promising capacity of DBS, hemorrhage and infections are associated risks, so the risks and benefits must be weighed out deliberately, and they must be communicated with the patient beforehand as well [65].

Computed tomography-derived fractional flow reserve (FFR-CT)

Computational fluid dynamics and anatomical data from CT scans are combined in FFR-CT for the non-invasive evaluation of coronary artery disease. This particular method has a possible impact on the diagnostic process and the management of vascular augmentation to stroke while being primarily used in the field of cardiology [66].

Focused ultrasound ablation

Thromboembolic stroke areas are being targeted by the cutting-edge non-invasive technique known as FUSA. By directing ultrasound waves precisely, FUSA, often combined with thrombolytic agents like tissue plasminogen activator (tPA), promotes thrombolysis and reperfusion, enhancing the treatment’s efficacy and safety [67]. Despite the promising results FUSA has shown, it is still in the process of clinical trials, and risks that are strongly associated with it include severe hemorrhagic complications when it is being used with tPA [68-70]. 

Comparative analysis of neurosurgical techniques

Whether the stroke is acute or chronic, its management requires a distinct approach tailor-made to specific requirements for each patient’s particular condition. In the case of acute stroke management, the main aim is the quick intervention to restore blood flow and eventually minimize brain damage. On the other hand, the management of chronic stroke majorly focuses on the re-establishment and prevention of further neurological decline. A comparative analysis of numerous techniques used in these settings has been described in this section (Tables 3, 4).

**Table 3:** Comparative overview of neurosurgical techniques in acute stroke management LVO, large vessel occlusion; MISTIE, minimally invasive surgery plus alteplase

Technique	Description	Key indications	Common complications
Endovascular clot retrieval	Mechanical thrombectomy using devices like stent retrievers and aspiration catheters	Acute ischemic stroke with LVO within 6-24 hours	Hemorrhage, reocclusion, and access site complications.
Neurosurgical clipping	Surgical placement of clips to seal off ruptured aneurysms	Aneurysmal subarachnoid hemorrhage	Rebleeding, brain edema, and infarction
Minimally invasive endovascular procedures	Techniques such as coiling, clot aspiration, and the MISTIE procedure for hemorrhage management	Intracerebral hemorrhage	Hemorrhagic complications and infections
Decompressive hemicraniectomy	Removal of part of the skull to relieve pressure from swelling brain tissue	Severe ischemic strokes with significant edema	CSF disturbances, seizures, and syndrome of the trephined

**Table 4:** Comparative overview of neurosurgical techniques in chronic stroke management EC-IC, extracranial-intracranial; DBS, deep brain stimulation; FUSA, focused ultrasound ablation

Technique	Description	Key indications	Common complications
Revascularization surgery	Direct (e.g., EC-IC bypass) and indirect (e.g., carotid endarterectomy) techniques to restore blood flow	Chronic ischemic strokes and significant artery stenosis	Stroke during surgery and myocardial infarction
Stereotactic surgery	Minimally invasive surgery using techniques like DBS and FUSA to target specific brain areas	Persistent motor deficits, pain syndromes, and epilepsy resulting from stroke	Hemorrhage, infection, and targeted area damage

## Conclusions

To conclude, the inspection of various techniques that are employed in the management of both acute and chronic stroke culminates it as a diverse and evolving field. Procedures such as MT are used for AISs to stereotactic surgeries and DBS, employed for chronic conditions, emphasize timely and accurate interventions as of particular importance. Advancements in the technology used for imaging and surgical procedures have profoundly enhanced the precision and harmlessness of these specific techniques, which subsequently allows for targeted treatment therapies that would improve patient outcomes and minimize potential risks. Nevertheless, appropriate patient selection, correct timing of the procedure, and multidimensional collaboration are all parameters on which the success of these interventions is majorly dependent. Continuous research and development in neurosurgical methods are of great importance as the burden of stroke is rising worldwide. This will ultimately result in improving our comprehension of cerebrovascular pathophysiology and broaden the range of therapeutic options available, which will lead to better management techniques and a higher standard of living for stroke survivors. This present thorough analysis emphasizes how crucial it is to incorporate cutting-edge innovation in neurosurgical techniques into the accepted stroke treatment protocols in order to maximize patient recovery and healthcare delivery while minimizing the risks.
